# 

**Published:** 1879-09

**Authors:** 


### CONTENTS.

### COMMUNICATIONS.

Address. *By Prof. C. N. Pierce* ........ 361
Dental Organizations. *By Prof. R. B. Winder* ........ 369
"Me and Him is Gradyates of the same College." *By W. H. Robinson, D. D. S., Suison, Cal* ........ 376

### PROCEEDINGS OF SOCIETIES.

Kentucky State Dental Association. *Reported by Dr. Chas. E. Dunn* ... 382

### EDITORIAL.

A Stumbling Block ........ 401
Transplanting ........ 403
Obituary ........ 404

